# *Fusobacterium nucleatum* and *Prevotella* in women with periodontitis and preterm birth

**DOI:** 10.4317/medoral.25874

**Published:** 2023-08-25

**Authors:** Karyne Martins Lima, Claudia Maria Coelho Alves, Flávia Castello Branco Vidal, Isaac Suzart Gomes-Filho, Jéssica Costa e Costa, Ricardo Della Coletta, Vandilson Pinheiro Rodrigues, Fernanda Ferreira Lopes

**Affiliations:** 1 Postgraduate Program in Dentistry (PPGO), Federal University of Maranhão, São Luís, Brazil; 2 Department of Morphology, Federal University of Maranhão, São Luís, Brazil; 3 Department of Health, State University of Feira de Santana, Feira de Santana, Bahia, Brazil; 4 Department of Oral Diagnosis, School of Dentistry, University of Campinas, Piracicaba, Brazil

## Abstract

**Background:**

Studies try to explain the hypothesis that maternal periodontitis may be associated with preterm birth.

**Material and Methods:**

This is a case-control study with 120, 40 cases (gestational age <37 weeks) and 80 controls (gestational age ≥37 weeks), that were submitted to the clinical periodontal examination and subgingival biofilm collection. Bacterial DNA of subgingival biofilm was performed and processed by qPCR.

**Results:**

Periodontitis was statistically significant in the Case group (35%) when compared to the Control group (11.2%) and Gingival Bleeding Index (GBI), sites with PS ≥ 4mm and sites with CAL ≥ 5mm were statistically higher in the Case group (*p* < 0.05). The proportions of *Pi* (*p* = 0.026) and *Fn* (*p* = 0.041) of subgingival biofilm were higher in the Case group. A greater number of sites with PS ≥ 4mm (r = -0.202; *p* = 0.026) and CAL ≥ 5mm (r = -0.322; *p* < 0.001) were correlated to lower gestational age.

**Conclusions:**

Periodontitis, preterm delivery, and/or low birth weight may have a possible relationship based on clinical parameters and the ratio of *Pi* and *Fn* at periodontal sites.

** Key words:**Periodontal diseases, premature birth, *fusobacterium nucleatum*, *prevotella intermedia*.

## Introduction

Defined as a multifactorial chronic inflammatory disease, periodontitis is associated with the presence of dysbiotic biofilm with a predominance of gram-negative anaerobic microorganisms and is mediated by the inflammatory response of the host (1). Periodontopathogens and their products can penetrate the bloodstream, reaching tissues of distant oral cavity structures. As a result, it can induce premature birth, preeclampsia, and cause or worsen cardiovascular diseases, low birth weight, aspiration pneumonia, and kidney diseases (2).

Many researchers have investigated the association between maternal periodontitis and premature birth and/or low birth weight babies (3,4) while there is research that shows that it is not possible to prove an association between the oral condition and adverse effects on pregnancy (5).

Although the exact mechanisms that clearly explain the participation of periodontitis in the adverse effects of pregnancy have not yet been established, some theories try to seek an explanation through two mechanisms: by the action of periodontal pathogens or by the effect of inflammatory mediators, such as interleukin-1 (IL-1), IL-6, IL-8, tumor necrosis factor-α (TNF-α) or prostaglandin E2 (PGE2), in the fetal-placental unit (6). Among the bacteria associated with periodontitis are the anaerobic gram-negative, Prevotela intermedia, and *Fusobacterium nucleatum* (7).

The presence of DNA from periodontopathogens has already been detected in the genital tract, amniotic fluid, and placental tissues, which supports how bacterial species of oral origin can participate in placental and intrauterine infections (8,9). Thus, the present study aimed to analyze whether there is a relationship between the clinical and microbiological parameters of periodontitis and the birth of premature babies, quantifying the pathogens*Prevotella intermedia (Pi)* and **Fusobacterium nucleatum* (Fn)* in the subgingival biofilm of postpartum women to verify if there is a relationship between the clinical and microbiological parameters of periodontitis and the birth of premature babies.

Materials and Methods

- Ethical Considerations

This research was approved by the Research Ethics Committee of the University Hospital of the Federal University of Maranhão under protocol number 002673/2011-60.

- Study design

A case-control study was carried out at the Maternal and Child Unit of the University Hospital of the Federal University of Maranhão, with puerperal women at the bedside attended up to 48 hours postpartum. Information about the mother's and newborn's health conditions were obtained from the medical records that were made available to the researchers at the time of collection.

The Case Group (A1) was formed by mothers of children born with a gestational age < 37 weeks and the Control Group (A2) by mothers of newborns with a gestational age ≥ 37 weeks.

Women smokers were excluded; alcoholics; those who used fixed orthodontic appliances; mouth breathers; chronic use of antibiotics or non-steroidal or steroidal anti-inflammatory drugs in the 6 months preceding the survey; the presence of a systemic condition that could influence the assessment (diabetes mellitus, systemic arterial hypertension, obesity, viral and bacterial infections); use of antihypertensive, anticonvulsant or immunosuppressant drugs or any other drug that is known to present the possibility of resulting in gingival tissue hyperplasia, twin pregnancy.

- Clinical Stage

A previously trained professional applied the questionnaire data collected on oral hygiene habits (performing or not brushing after meals and its frequency, using or not flossing and its frequency, and whether you visited the dentist in the last year). The clinical examination was performed with the aid of a frontal light attached to the examiner's head. The following parameters were evaluated: 1- Probing Depth (PS) recorded at 6 sites for each tooth (mesiobuccal and mesiolingual, distobuccal and distolingual and in the mid-buccal and mid-lingual region), 2 - Clinical Attachment Level (CAL) distance in millimeters between the cementoenamel junction and the bottom of the sulcus or periodontal pocket (6 sites), 3- Visible Plaque Index (VPI) (buccal, mesial, distal and lingual), 4- Gingival Bleeding Index (6 sites), recorded 10 seconds after probe removal North Carolina PCPUNC 156 (Hu-Friedy, Chicago, USA) (Hu-Friedy, Chicago, USA) of the periodontal pocket or sulcus (10,11).

Each postpartum woman was classified according to the presence or absence of periodontitis according to the following criteria: at least 4 or more teeth with one or more sites with PS ≥ 4 mm, with CAL ≥ 3 mm in the same site, and the presence of bleeding on probing (12).

- Biofilm Collection

The collection of the subgingival biofilm was performed after the removal of the supragingival biofilm with sterile gauze. Samples were taken with Gracey 5-6 / 7-8 periodontal curettes (Hu Friedy, Chicago, USA) from four different sites (those with the greatest probing depth of each quadrant) throughout the same depth. The biofilms were placed in sterile tubes containing a Tris-EDTA solution (150 µL Tris-EDTA + 100 µL of NaOH solution) for freezing in a -80°C freezer (Indrel Ultra Freezer -80°C).

- DNA Extraction

DNA extraction was performed as described by the QIAamp DNA User Manual (Qiagen), with modifications. 200µL of the sample, 20µL of proteinase K and 200µL of Buffer AL were added to a 2ml tube and incubated at 56°C for 10 minutes in a dry bath. Then 200µL of ethanol (96-100%) (Vetec Química, Brazil) was added and the entire solution was added to a silica column for DNA capture.

500µL of washing solutions were added followed by centrifugation and finally, the column was incubated in 200µL of buffer AE for 5 minutes. DNA was collected after centrifugation at 8000rpm for 1 minute. The purified DNA was submitted to quantification in the Nanovue device (GE, USA) to evaluate the concentration and integrity of the DNA. All material was frozen in -80°C frfreezerIndrel Ultra Freezer -86°C).

- Detection and Quantification by Real-Time Polymerase Chain Reaction (qPCR)

For the detection and quantification of the bacteria under study, the DNA of the bacteria needs positive controls, as these are necessary to generate the standard curves of the bacterial species used in the biofilm model. Concentrations of 1-0.001ng of DNA were defined for the standard curve generation. The logarithm of the corresponding values ​​from the quantification cycle was used to obtain a linear regression. For the quantification of bacteria in the biofilm samples, the concentration of the extracted DNA mixture was determined using the NanoDrop ND-1000.

After defining the standard curve, qPCR of the DNA of the samples was performed with the 16S ribosomal RNA gene primer of all the bacteria to control the quality of the sample's DNA. The specific PCR primers*Prevotella intermedia (Pi)* (5’-TTTGTTGGGGAGTAAAGCGGG-3’) and **Fusobacterium nucleatum* (Fn)* (5’-ATT GTC GCT AAA AAT T -3’) were used (13).

All reactions were quantified individually in separate wells for each bacterial species and performed in triplicate. The qPCR was performed in a total reaction volume of 20 µL, containing 10 µL of SYBR® Green PCR Master Mix (Life Technologies, Zug, Switzerland), 5 µL of the sample (diluted to contain 1 ng of DNA), and 5 µL of solution of primer from the respective bacterium (10µM, a mixture of 3' and 5' primers).

Amplification took place in a StepOne thermocycler (Applied Biosystems) by initial incubation of 2 min at 50 °C and 10 min at 95 °C, followed by 40 cycles of 15 s at 95 °C and 1 min at 60 °C. Ct obtained, the DNA concentration of the sample was calculated for each organism using the theoretical genomic weight of each of the bacteria (13).

- Statistical analysis

Data were analyzed using GraphPad Prism version 8 software (GraphPad Software, San Diego, USA). Descriptive statistics were performed using measures of frequency, mean and standard deviation. The results were presented through Tables, bar graphs, and ≥heat maps.

Categorical variables were compared between case and control groups using the chi-square or Fisher's exact test. Normality analysis of continuous variables was performed using the Shapiro-Wilk test. After this procedure, the independent student t-test was selected for the comparative analysis between the groups. In addition, Pearson's coefficient (r) was used to analyze the correlation between the variables gestational age (in weeks), the proportion of pathogens, and periodontal parameters. The significance level adopted for all analyzes was 5% (*p* < 0.05).

## Results

The birth weight of the Case group was statistically lower compared to the Control group (*p* < 0.001). The Case group also observed a lower mean number of prenatal consultations (*p* = 0.045). There was a concentration of younger women in the Control group (*p* = 0.014). Furthermore, arterial hypertension was more prevalent in the Case group (*p* < 0.001) (Table 1).

The evaluation of the periodontal condition, expressed in Table 2, revealed that the prevalence of periodontitis was statistically significant in the Case group (35%) when compared to the Control group (11.2%). It was also observed that the parameters of Gingival Bleeding Index (GSI), sites with PS ≥ 4mm, and sites with CAL ≥ 5mm were statistically higher in the Case group (*p* < 0.05).

**Table 1:**
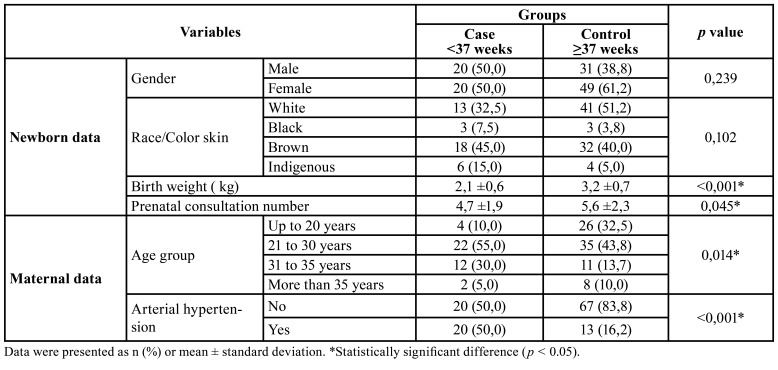
Distribution of general characterization variables between groups.

**Table 2:**
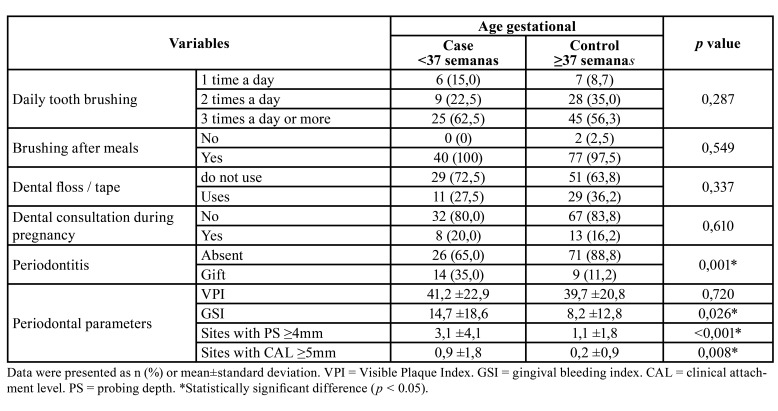
Comparative analysis of oral hygiene variables and periodontal condition between groups.

**Figure 1:**
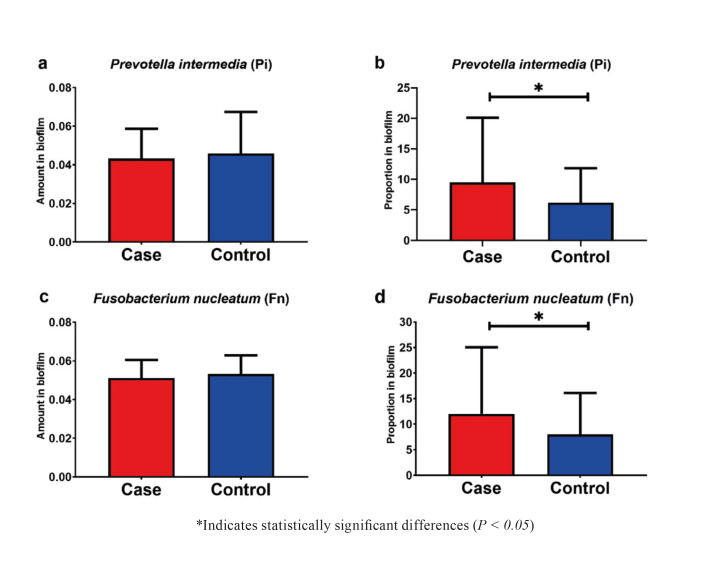
Comparative analysis of the amount and proportion in the biofilm of the pathogens *Prevotella intermedia* (a and b) and *Fusobacterium nucleatum* (c and d).

Fig. 1 illustrates the distribution of pathogens in the samples evaluated. There were no significant differences between the groups when comparing the total amount of *Pi* and Fn. On the other hand, the analysis of the proportion of pathogens in the samples revealed that the proportions of *Pi* (*p* = 0.026) and *Fn* (*p* = 0.041) were higher in the Case group.

**Figure 2:**
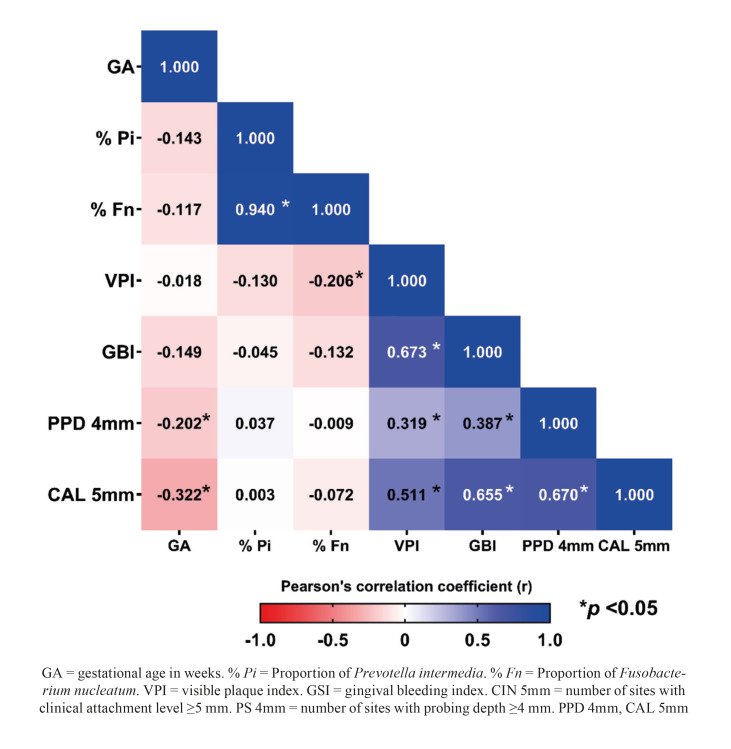
Pearson correlation analysis between gestational age (in weeks), pathogens and periodontal variables.

Linear correlation analysis is illustrated in the heat map (Fig. 2). The results showed that the greater the number of sites with PS ≥ 4mm (r = -0.202; *p* = 0.026) and CAL ≥ 5mm (r = -0.322; *p* < 0.001), to lower the gestational age. The proportions of *Pi* and *Fn* showed a strong direct\ correlation (r = 0.941; *p* < 0.001). The VPI showed an inverse correlation with the proportion of *Fn* (r= -0.206; *p*= 0.023). In addition, all periodontal parameters showed significant direct correlations with each other.

## Discussion

The study showed the relationship between periodontitis and preterm delivery and/or low birth weight, through clinical and microbiological parameters of periodontal disease. It is noteworthy that the analysis of the proportion of pathogens in the samples revealed higher proportions of *Pi* and *Fn* in postpartum women with preterm delivery.

It is noteworthy that in both groups there were no significant differences in the total amount of *Pi* and *Fn* between groups, however, the proportion of *Pi* (*p*=0.026) and *Fn* (*p*=0.041) within the universal DNA community of the subgingival biofilm of postpartum women were higher in the case group. Data partially corroborate another case-control study with pregnant women in which the results found an association in the clinical presentation of periodontitis, but in the microbial analysis was no significant association between preterm delivery and low birth weight with most periodontal pathogens (14). A possible explanation for this divergence may lie in the method used for microbial analysis by PCR (15) that detects only presence or absence and by qPCR, as in the present study.

Another relevant aspect is the methodology presented here by the use of the 16S universal primer, which allowed the identification of bacterial phenotypic sequence present in most bacteria, with subsequent use of a bacterial primer specific to the *Fn* and *Pi* pathogens (16). This fact clarifies that the total amount of *Pi* and *Fn* is not significant between the two groups (case-control), however, the analysis of the proportion of pathogens in the samples revealed higher proportions of *Pi* and *Fn* in the group with preterm and/or low delivery birth weight.

Case-control studies have associated the presence of oral pathogens, including *Fn* and Pi, with hypertensive disorders during pregnancy, as they have been shown to have higher levels in hypertensive women than in normotensive patient groups (9,17). In the present study, it was found that there were more hypertensive mothers among the case group participants, a situation that corroborates the literature on the birth of premature and/or low birth weight babies (8). In addition, high levels of bacteria and viruses in the subgingival and placental samples occur more in women with preeclampsia and periodontitis compared to women with preeclampsia and without periodontitis (17).

The number of prenatal consultations was lower in the case group and this data is similar in part to the findings of Martínez-Martínez *et al* (18) who carried out a study to identify pathogens in biofilms by PCR. They concluded that the main periodontal bacteria are not related to premature birth, but that mothers in the case group show less care for hygiene and health, such as lower number of prenatal consultations, higher level of supragingival biofilm, lower toothbrushing frequency, higher levels of active caries and more need for dental treatment. Thus, they suggest that preterm birth would be multifactorial and the only presence of periodontal bacteria would not be able to trigger it (18).

Fn is one of the most prevalent oral species related to adverse effects in pregnancy (19). It has been detected in a wide variety of placental and fetal tissues, including amniotic fluid, fetal membranes, umbilical cord blood, neonatal gastric aspirate, fetal lung, and stomach, associated with chorioamnionitis, preeclampsia, preterm birth, stillbirth, and early neonatal sepsis (19,20).

Periodontitis is attributed to interactions between host immunity and multiple microbial species, including *P. *gingivalis**, *F. nucleatum*, *P. intermedia*, *A. actinomycetemcomitans*, T. denticola, in the subgingival environment, and the DNA of periodontal pathogens is highly detecTable, in patients with periodontitis compared to controls (16).

To date, the study by Doyle *et al* (21) can be considered the largest in the number of participants with more than 1000 individuals in the placental microbiota analysis, which found bacterial DNA in more than 50% of placental tissues, revealing differences between the oral and vaginal microbiota being the vaginal microbiota with the highest association with adverse effects on pregnancy in their findings. However, it is noteworthy that periodontal pathogens were detected simultaneously in pregnant women's subgingival biofilm and the placenta (22).

The periodontal clinical and microbiological parameters of postpartum women showed periodontitis as a factor associated with preterm birth, and these mothers had babies with lower weights compared to the control group. Some studies (23-26) reported an association between periodontitis and preterm birth or low birth weight, while others found no association. The different results may be explained by the use of different parameters for the diagnosis of periodontitis as well as the use of different terms: premature newborn, low birth weight, or premature and low birth weight (27).

It is important to highlight that a systematic review of 10 case-control studies concluded that there is a relationship between periodontal disease and increased risk of preterm birth and low birth weight or low birth weight with Odds Ratios ranging between 2.04 and 4.19, and in this work, there was only one study that found no association (28). Thus, the results presented here such as the GSI parameters, sites with PS ≥ 4mm, and sites with CAL ≥ 5mm, which were statistically higher in the Case group (*p* < 0.05) support the relationship between periodontitis and adverse effects of pregnancy.

For Fischer *et al* (29), it is premature to rule out the association between periodontitis and adverse pregnancy outcomes by studies that showed failure in periodontal treatment to reduce adverse pregnancy outcomes. One possible hypothesis is that periodontal pathogens spread to the placental tissue before periodontal treatment. It is important to consider that the association must be related to the presence of oral pathogens in the placenta and the amount, prevalence, and exposure time.

Other studies also sought answers to possible failures in the work to explain the non-association between periodontitis and adverse effects on pregnancy, such as heterogeneity in the clinical definition of the severity and extent of periodontitis used to distinguish case and control groups. Studies fail to control for shared risk factors or confounding factors between periodontitis and adverse effects on pregnancy, and finally, most studies did not consider the spread and survival of periodontal pathogens to the placenta as a mechanism that could induce these effects regardless of ongoing disease in the oral cavity (30).

This research presents strong bridges such as the criteria used for the diagnosis of periodontitis, the sample size respecting the case and control groups, and the robust technique for detecting periodontal pathogens through qPCR. The findings contribute to the hypothesis of an association between *Pi* and *Fn* bacteria and the prematurity outcome, however, we were unable to show a causal relationship.

## Conclusions

Periodontitis and preterm delivery may have a possible relationship based on clinical parameters and the ratio of *Pi* and *Fn* at periodontal sites, contributing to the multifactorial nature of preterm delivery. However, we cannot assert forcefully the cause-and-effect relationship between the objects studied in the research. Thus, more studies are needed to corroborate the preliminary data of the present study.
